# State-of-the-Art Review on Engineering Uses of Calcium Phosphate Compounds: An Eco-Friendly Approach for Soil Improvement

**DOI:** 10.3390/ma15196878

**Published:** 2022-10-03

**Authors:** Maksym Avramenko, Kazunori Nakashima, Satoru Kawasaki

**Affiliations:** 1 Division of Sustainable Resources Engineering, Graduate School of Engineering, Hokkaido University, Sapporo 060-8628, Japan; 2 Division of Sustainable Resources Engineering, Faculty of Engineering, Hokkaido University, Sapporo 060-8628, Japan

**Keywords:** novel grout material, soil improvement, sustainable geotechnics, calcium phosphate compounds (CPCs), calcium phosphate precipitation

## Abstract

Greenhouse gas emissions are a critical problem nowadays. The cement manufacturing sector alone accounts for 8% of all human-generated emissions, and as the world’s population grows and globalization intensifies, this sector will require significantly more resources. In order to fulfill the need of geomaterials for construction and to reduce carbon dioxide emissions into the atmosphere, conventional approaches to soil reinforcement need to be reconsidered. Calcium phosphate compounds (CPCs) are new materials that have only recently found their place in the soil reinforcement field. Its eco-friendly, non-toxic, reaction pathway is highly dependent on the pH of the medium and the concentration of components inside the solution. CPCs has advantages over the two most common environmental methods of soil reinforcement, microbial-induced carbonate precipitation (MICP) and enzyme induced carbonate precipitation (EICP); with CPCs, the ammonium problem can be neutralized and thus allowed to be applied in the field. In this review paper, the advantages and disadvantages of the engineering uses of CPCs for soil improvement have been discussed. Additionally, the process of how CPCs perform has been studied and an analysis of existing studies related to soil reinforcement by CPC implementation was conducted.

## 1. Introduction

**Figure 1:**
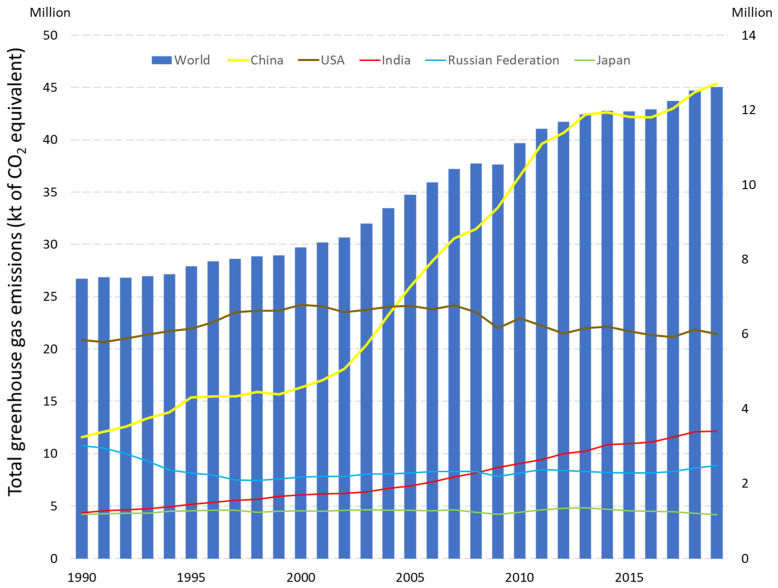
Representation of the total greenhouse gas emissions in the world, China, USA, India, Russian Federation, and Japan. Bar chart shows the emissions worldwide, line graphs show the emissions in China, USA, India, Russian Federation, and Japan with appropriate values on the left and on the right, respectively (based on online databases in World Bank Data [8]).

Climate change is recognized as one of the most important environmental issues worldwide [1]. According to the World Bank Data, greenhouse gas emissions have risen dramatically since 1990 from 29.4 Gt in the beginning to 46.3 Gt in 2019 and are continuing to rise. Figure 1 shows the uptrend in carbon dioxide (CO_2_) emissions globally and in each of the world’s leading five countries by CO_2_ emissions. China is the country with the highest level of CO_2_ emissions and has a rapidly increasing tendency. According to 2019 data, China is responsible for 12.7 Gt, USA 6 Gt, India 3.4 Gt, the Russian Federation 2.5 Gt, and Japan 1.2 Gt of CO_2_ emissions. This rapid upward trend in greenhouse emissions is directly related to cement production worldwide and the demand for cement due to increasing globalization. It is considered the second most consumed material in the world next to water [2]. Cement consumption in 2021 reached 4.4 billion tons [3], with carbon dioxide (CO_2_) emissions from its production being 450 kg/m^3^ [4], which is responsible for 25% of worldwide manufacturing [5] and 8% of global human-generated CO_2_ emissions [6]. The predicted emissions from cement production worldwide is projected to reach 2.34 billion tons of CO_2_ in 2050 [7].

In order to reduce the demand for cement and maintain the rate of industrialization, it is necessary to improve the conventional soil improving methods to make them environ-mentally friendly or invent new ones. This requires investigating and improving construction and engineering concepts for soil improvement in an eco-friendly way, which will lead to less pollution and a zero-carbon footprint in the future.

As a viable alternative to standard methods of soil improvement, environmentally friendly, eco-methods, which involve the activity of microorganisms and/or their by-products, have gained the uttermost popularity.

Biomethods are alternatives to conventional methods and can completely replace them for filling and grouting materials with small pores. These methods can replace conventional ones for specific geotechnical purposes such as soil improvement before construction, stabilization of slopes and dams, stabilization of sandy soil, prevention of wind and water erosion, for waterproofing ponds, canals, landfills, reservoirs as well as for chemical, radioactive, and biological soil immobilization [9].

Despite the fact that they are excellent candidates for widespread application, at this stage, these technologies have some negative consequences that will be discussed in this review, and novel material will be introduced.

## 2. Existing Methods of Soil Improvement

Ground improvement techniques can be categorized into mechanical modification (physical manipulation), hydraulic modification (drainage or dehydration), physical and chemical modification (introducing materials or chemicals into soil), and modification by inclusions, confinement and reinforcement (using of structural members) [10].

One of the main purposes of improving soil properties is to increase the strength, stiffness and stress in its stable state by the filling and compaction of voids (air, water). The use of calcium-based cements or their analogues for this purpose is the most common [11].

Based on the ecological aspect, soil improvement techniques can be classified into the following two groups: conventional and ecological. Conventional methods are those that have been used in engineering for a considerable period of time since they are widely known, cheap, and well-researched. In contrast, ecological methods are methods that have no direct impact on the environment in their production or application, are often novel, and require broad publicity and investigation.

### 2.1. Conventional Methods

Conventional soil improvement methods can be characterized into two main groups: (i) mechanical compaction and (ii) the injection of cement or other binding agents into the soil. While the former is more energy-intensive, the latter requires the use of binding agents that can be harmful to the environment and human health [12].

Mechanical techniques involve extrusion and substitution, stepped structures, pre-loading, a masonry column method, nailing to the ground, and synthetic armoring applications [13]. Binding agents can be introduced into the ground and combined with present geomaterials. The following substances can be applied to the soil: cement, slag, lime, silicate-based gel, fly ash, and a wide variety of other agents for modifying the geotechnical properties of the soil. Altering the soil properties with these types of treatments is more effective in maintaining improvements over the long-term than other methods [14,15].

As a result of the widespread use of traditional Portland cement, conventional methods for soil enhancement are primary carbon dioxide emitters globally [16].

Hence, eco-friendly practices are the most preferable methods to improve the soil at the present time and in the future.

### 2.2. Ecofriendly Methods

In 2006, the American Society of Civil Engineers (ASCE) acknowledged the leadership role of engineers in promoting sustainability and their role in ensuring both quality and innovation in addressing sustainability issues [17]. Therefore, many countries have been researching the matter of sustainable development and ecofriendly methods. 

The most studied and widely applied ecofriendly methods for soil improvements by far are microbial-induced carbonate precipitation (MICP) and enzyme induced carbonate precipitation (EICP). These methods use microorganisms and urease, respectively, to precipitate calcium carbonates.

#### 2.2.1. MICP

**Figure 2:**
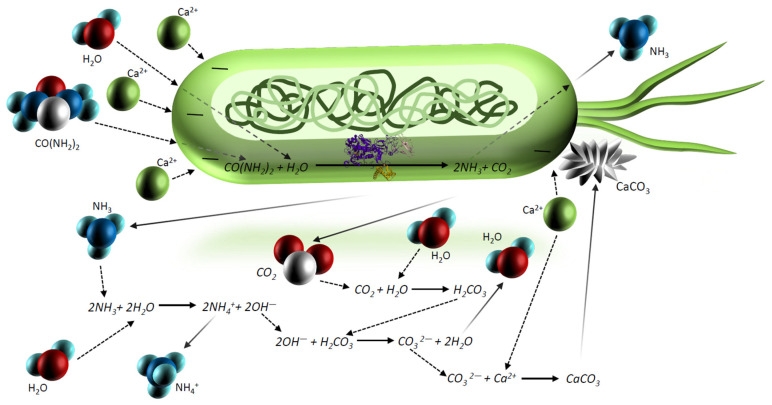
A representation of calcite precipitation due to the MICP method.

MICP is a technique that has gained tremendous popularity and involves the precipitation of carbonate due to the activity of microorganisms. This method is based on the enzymatic activity of microorganisms that breaks down urea into carbonate and ammonium ions (Figure 2). It was first discovered by Bouquet in 1973, who explored the formation of crystals by soil bacteria cultivated on solid media [18]. However, the term “microbe-induced calcium carbonate precipitation” itself was not coined until 2004 by Wiffin [19,20].

Appropriate bacteria for initiating biocementation need to be non-toxic, mineralization tolerant, and resilient to survive in the present soil environment [21]. Microorganisms of natural origin that can induce calcium carbonate precipitation include cyanobacteria, sulfate-reducing bacteria, methane oxidizing bacteria, denitrifying bacteria, ammonifying bacteria, and the most studied, urease-producing bacteria [4,19,22]. The most suitable bacteria for MICP to date are ureolytic bacteria, particularly, *Sporosarcina pasteurii*; due to the electronegativity of their walls, the bacteria easily attaches to soil particles and can provide nuclear sites for calcite precipitation [23].

Various factors affect the calcium carbonate formation pathway and bacterial performance including the type of bacteria, concentration of microorganisms, calcium ratio, presence of nucleation sites, enzymatic activity, temperature, grouting method, injection flow rate, pH, presence of nutrients, and calcium ions in the environment [4,19,21,22,24]. Different species of bacteria require different conditions for growth and reproduction, but for most, the optimal conditions are as follows: temperature 20–37 °C, pH 6.5–9, curing time—14 days, concentrations of urea and calcium ion—0.5 mol/L, injection frequency—once per day [4,19,21,22,25,26].

In the presence of urea, $\mathrm{CO}{\left({\mathrm{NH}}_{2}\right)}_{2}$, in solution, bacteria start producing carbon dioxide (${\mathrm{CO}}_{2}$) and ammonia (${\mathrm{NH}}_{3}$), as a by-product (Equation (1)). At the same time, ammonium ions (${\mathrm{NH}}_{4}{}^{+}$), hydrogen ions (${\mathrm{OH}}^{-}$), and carboxylic acid ($H_{2}{\mathrm{CO}}_{3}$) are produced by the dissolution of ammonia (${\mathrm{NH}}_{3}$) and carbon dioxide (${\mathrm{CO}}_{2}$) in water, respectively, which leads to an increase in the pH of the environment (Equations (2) and (3)). Under alkaline conditions, carboxylic acid ($H_{2}{\mathrm{CO}}_{3}$) reacts with hydroxide ions (${\mathrm{OH}}^{-}$) to form carbonate ions (${\mathrm{CO}}_{3}{}^{2-}$) (Equation (4)), which consequently react with calcium cations (${\mathrm{Ca}}^{2+}$) and precipitate calcium carbonate (${\mathrm{CaCO}}_{3}$) (Equation (5)).

(1) $$\mathrm{CO}{\left({\mathrm{NH}}_{2}\right)}_{2}+{\;H}_{2}O\;\overset{\mathrm{microbial}\;\mathrm{urease}}{\to }2{\mathrm{NH}}_{3}+{\mathrm{CO}}_{2}$$

(2) $$2{\mathrm{NH}}_{3}+2H_{2}O\;\overset{\;}{\to }2{\mathrm{NH}}_{4}{}^{+}+2{\mathrm{OH}}^{-}$$

(3) $${\mathrm{CO}}_{2}+{\;H}_{2}O\overset{\;}{\to }H_{2}{\mathrm{CO}}_{3}\;$$

(4) $$2{\mathrm{OH}}^{-}+{\;H}_{2}{\mathrm{CO}}_{3}\overset{\;}{\to }{\mathrm{CO}}_{3}{}^{2-}+2H_{2}O$$

(5) $${\mathrm{Ca}}^{2+}+{\;\mathrm{CO}}_{3}{}^{2-}\overset{\;}{\to }{\mathrm{CaCO}}_{3}\;$$

The process of MICP is briefly outlined in Equation (6). One mole of $\mathrm{CO}{\left({\mathrm{NH}}_{2}\right)}_{2}$ undergoes hydrolysis and reacts with ${\mathrm{Ca}}^{2+},$ resulting in the formation of two moles of ${\mathrm{NH}}_{4}{}^{+}$ and one mole of ${\mathrm{CaCO}}_{3}$.

(6) $$\mathrm{CO}{\left({\mathrm{NH}}_{2}\right)}_{2}+{\;H}_{2}O+{\;\mathrm{Ca}}^{2+}\;\overset{\;}{\to }2{\mathrm{NH}}_{4}{}^{+}+{\mathrm{CaCO}}_{3}$$

Below is a summary of the advantages and limitations of MICP.

##### Advantages of MICP

- MICP can solve a range of important geotechnical and environmental challenges such as soil reinforcement, reducing the risk of ground degradation to landslides, preventing liquefaction during earthquakes, stabilizing oil, the production of bio-concrete materials, heavy metal and radionuclide retention, sewage treatment, concrete repair, the modification of mortar, tunnel wall stabilization, enhance oil recoverability and reservoir profile control as well as water plugging, CO_2_ capture, and storage [4,19,22,23,27].
- This method is superior to conventional methods since traditional methods are more complex in terms of construction and are time-consuming, energy-intensive, and low productive [28].
- Applying this method allows for an to increase in the unconfined compressive strength (UCS) of the soil of up to 12.4 MPa [23,29].
- By applying some species of bacteria to the soil, atmospheric CO_2_ levels can be reduced. These bacteria do not produce nitrogen-based by-products, making them more environmentally friendly [19]. The following bacteria have the potential to be used: sulfate-reducing bacteria increases the transformation rate of CO_2_ into solid minerals [30], cyanobacteria during MICP absorb CO_2_ from the air and utilize it to precipitate CaCO_3_ [31], and bacillus mucilaginous produces carbonic anhydrase, which can remove CO_2_ from the air to precipitate CaCO_3_ [32].
- Unlike conventional methods, MICP leaves the soil structure unaffected throughout the entire period of treatment [23].

##### Limitations of MICP

**Figure 3:**
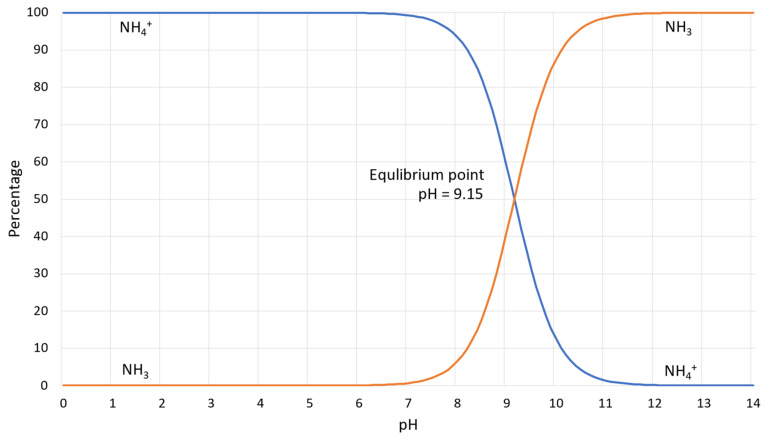
Dependence of percentages of free ammonia ($NH_{3}$) and ionized ammonia (ammonium ($NH_{4}{}^{+}$)), abundance, and pH at T = 25 °C [33].

- Ammonium and ammonia produced by the enzymatic reaction are dangerous and harmful substances, and in large concentrations, causes toxic effects on human health and has impacts on the flora and deposition of nitrogen in the atmosphere [12,21,24]. Figure 3 illustrates the percentage dependence of free ammonia and ionized ammonia in solution when the pH is changed from 0 to 14 at 25 °C. According to Figure 3, under pH 6, almost 99% of all ammonia remains in ionized form, whereas after pH 7, it rapidly converts to free ammonia and contaminates the environment. At pH 7.15, the percentage of ammonia passes the 1% level, and increases rapidly after pH 8, reaching the equilibrium point of 50% at pH 9.15. At pH 11.15, the percentage of ammonium passes the 99% level and the increase slows down considerably.
- The reaction pathway is slower and more complicated than in the case of chemicals [21].

- The CaCO_3_ deposition is not uniform and most of it is deposited near the injection point, which consequently leads to the appearance of bioplugging, the phenomenon of the inability to penetrate fine sandy and silty soils due to clogging in the void space in the upper layers of the soil [25,34,35].
- This method is costly to implement in the open field. The primary cost of MICP is represented by bacteria and nutrients, which leads to higher costs (from 25–75 $US/m^3^ to about 500 $US/m^3^) [12,22].
- MICP is limited to large-grain materials because of the size of the bacteria, most of which is between 0.5 μm to 3 μm in size (*S. pasteurii* has a cell size of around 2800 nm) [17,34].

#### 2.2.2. EICP

**Figure 4:**
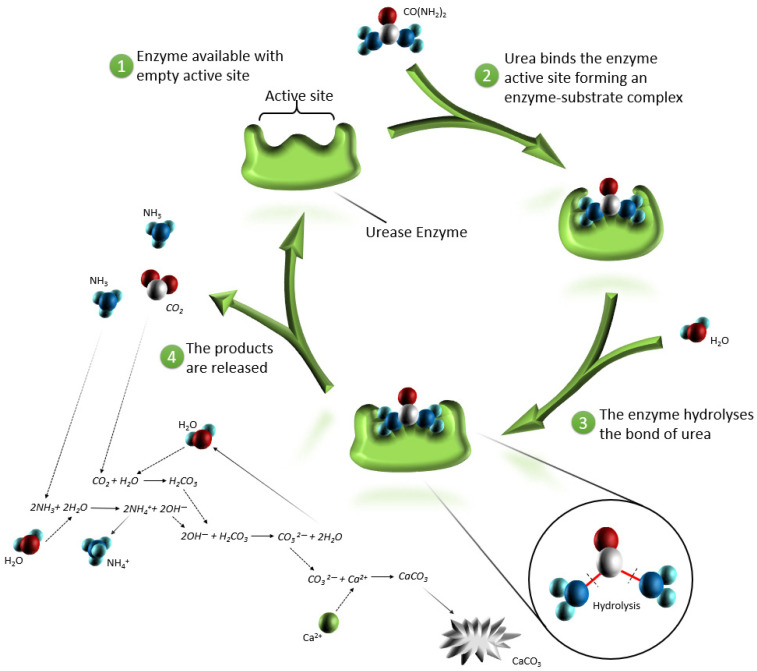
A representation of calcite precipitation due to the EICP method.

The term EICP was first introduced by Kavazanjian in 2015 [36]. This technique, similar to MICP, is a bio-cementing technology, but in contrast to MICP, it involves enzymes isolated from bacterial or plant solutions rather than bacteria (Figure 4) [29]. In 1926, James B. Sumner was the first who obtained a new protein (urease) from beans that can degrade urea into ammonium and carbonate [37]. However, it was only in 2003 that Nemati and Voordouw first investigated the enzymatic generation of CaCO_3_ in porous environments [38], and in 2011, an enhancement in the mechanical and hydraulic characteristics of the sand samples was shown by Yasuhara [39]. Urease is a nickel-dependent metalloenzyme that can be naturally obtained by plants, bacteria, fungi, and algae [40]. The jack bean plant, *Canavalia ensiformis*, is the most extensively studied source of enzymes for application in EICP [41,42].

Urease contains nickel ions, which are essential for the hydrolysis of urea. These metal ions on the active sites of urease catalyze the reaction. During calcite deposition, urease undergoes many hydrolysis cycles until the nickel source is completely consumed [43]. In contrast to the bacterium, which loses access to oxygen and dies as a result of being enclosed by calcite, the urease has no such disadvantage [44].

The efficiency of EICP depends on the origin of the enzyme, the activity of the enzyme, the enzyme and calcium concentration in solution, the pH and temperature of the environment and the method of EICP treatment [16,25]. According to the literature, the optimal conditions could be as follows: temperature 25–30 °C, pH of 7–9, concentration of urea 0.5 M, concentration of urease enzyme 1–3 g/L, and a curing time of 7–14 days [42,45,46].

This method has been applied to the following issues: improvement in soil strength, wind erosion prevention, reduction in permeability, sand softening control, reduction in flying dust exposure, bio-brick manufacture, surface water erosion control, restoration of contamination by heavy metals, desert sand stabilization, and the repair of fractures in concrete [16,42,45].

Below is a summary of the advantages and limitations of EICP.

##### Advantages of EICP

- The urease molecule has a size of 12 nm, allowing this method to be superior to MICP and can be used to stabilize fine-grained and highly compacted soils with voids with sizes less than 0.5 μm [25,34,41]. In addition, they are highly soluble in water, thus perfectly injectable into the soil [47].
- The UCS of soils treated with EICP can reach 6.5 MPa [16].
- EICP requires less monitoring than MICP and is less energy consuming [41].
- Urease extracted from plants can be a cost-effective alternative to chemical purified urease [34].
- In comparison to the MICP method, the EICP method is biologically safer since it does not involve bacteria [48].

##### Limitation of EICP

- The cost of EICP is prohibitive because 57–98% of the cost of the treatment solution comes from the enzyme urease [49]. The price of urease from *Canavalia ensiformis* (Jack bean) is 12.8 US $/g with a urease activity of 1 U/mg [50]. According to our approximate calculations, the use of urease in the open field will cost about 100,000 to 200,000 $US/m^3^.
- Similar to MICP, EICP produces ammonium as a by-product, which is toxic for humans and hazardous to the aquatic and atmospheric environment, in addition, due to the high pH level as a consequence of this method, the risk of corrosion is increased [16,27].
- Using the enzyme instead of bacteria results in the removal of binding points, potentially reducing the efficacy of the method and the strength [16].

According to the Japanese Uniform National Wastewater Standards, the limit for ammonia in a ground environment is 100 mg/L [51]. Estimations using Equations (1)–(5) demonstrated that approximately 10.5 kg of ammonia will be released into the atmosphere and 11.2 kg of ammonium will be emitted into an aqueous environment during the biocementation of 1 m^3^ of sand [52]. This amount of pollution in proportion to the volume of the sand is equivalent to a concentration of 10,500 mg/L and 11,200 mg/L for ammonia and ammonium, respectively, which is more than 100 times higher than the limit. Therefore, to ensure that biocementation methods meet the requirements, it is necessary to reduce the amount of ammonia produced by at least 99.9%. Based on this, it can be concluded that MICP/EICP techniques should not be used in open field experiments without supporting environmental protection technologies [9].

In previous studies [53,54,55,56,57,58,59,60,61], a variety of methods have been used to reduce the ammonium from soil. The most effective method for removing ammonia is the flushing/rinsing method with a reduction rate of 99.8% in aqueous NH_4_^+^. When using a flushing solution (200 mM CaCl_2_, pH ≈ 10.0) with both high pH and ionic intensity with MICP, the final NH_4_^+^ concentration in the soil samples was significantly reduced by 90.6–99.8% [61].

This is followed by the MISP technique (microbially-induced precipitation). This method follows the same process as MICP, but due to the presence of soluble phosphate and magnesium ions in the solution, the NH_4_^+^ that forms during the urease hydrolysis process is bound to magnesium-ammonium phosphate (struvite MgNH_4_PO_4_·6H_2_O). Using this method, the ammonia gas emissions can be reduced in the range of 75.12% to 97.79% [53,54,55].

The use of zeolite is a good candidate for the reduction in ammonium ions. Zeolite is a natural material that has a high cation exchange capacity and an excellent affinity for ammonium. In the presence of this mineral, free ammonium ions are bound, thereby absorbing up to 75% and 43% of the maximum theoretical concentration for EICP [56] and MICP [57], respectively.

Another approach to solve the ammonium problem is to use different types of bacteria and another source of calcium. By adding the Gram-positive lactic acid bacterium *Pediococcus acidilactici*, 38% of emission reductions can be achieved [58]. It has been proven that the use of calcium acetate as a calcium source for MICP could reduce NH_3_ emissions by 54.2% [59].

Using a low-voltage (35 Volts) electric field to capture ammonium after MICP revealed promising prospects. In this method, solar panels were used to generate electricity and an anode and cathode to control the MICP reaction, and eventually bind the ammonium ions. This technique allows for the prevention of the leakage of NH_4_^+^ into the soil by capturing it in the cathode chamber by the graphite cathode electrode [60].

## 3. Calcium Phosphate Compounds

None of the above-mentioned techniques are capable of reducing all of the ammonium emissions to the atmosphere and soil when MICP/EICP is applied. Potentially, the combined application of several techniques could be effective and obtain a level below the standard, but it might not be economically justified. Therefore, it is necessary to use other techniques acceptable for field applications. One of the potential techniques is calcium phosphate precipitation. This concept is relatively new; however, in recent years, there has been a clear upward trend in the popularity of this topic.

### 3.1. Mechanisms of Soil Improvements Using CPCs

Calcium phosphate compounds (CPCs) are highly biocompatible materials consisting mainly of calcium phosphate and can rapidly harden into a solid mass through a self-hardening mechanism [62,63].

The scientific literature on the use of calcium phosphates for bone repair dates back to the 1920s [63]. Hydroxyapatite has been manufactured synthetically since the early 1970s and applied in clinical practice as a main component of self-hardening calcium phosphate compounds from the beginning of the 1980s [64]. Hydroxyapatite is an essential component of bone tissue and teeth, making it an excellent and affordable source for CPCs [65]. Calcium phosphate materials have been used for decades in medicine and dentistry in the form of cements, composites, and coatings [66]. In 2010, they were introduced for soil enhancement (Table 1).

CPCs can be divided into two main categories, depending on the course of the setting reaction [63]:

i. Reaction between calcium phosphate compounds alone

In this type of reaction, only one precursor compound of Ca and P mixes with the liquid phase, leading to the hydrolysis and precipitation of CPCs [67]. Different conditions are required for the deposition of different phases of calcium phosphate. The pH of the medium and the atomic ratio of calcium and phosphorus have a major influence on the course of the reaction. To control the calcium-phosphorus ratio, different calcium and phosphorus resources and concentrations could be used. To control and increase the pH of solution, microorganisms [68,69,70] or ureases [71,72,73] can be used. However, CPCs precipitation is possible even without pH control [74,75,76,77,78,79,80].

ii. Reaction between calcium and carboxylic acid

Acid–base interaction requires several Ca and P precursor compounds and acid that react with each other and produce a neutral final product [67].

Calcium phosphate precipitation typically involves the use of organic acids to improve its mechanical properties and setting characteristics [81]. Organic acids are able to bind calcium ions and interact with growing CPC crystals while slowing down rapid setting. Carboxylic acids without hydroxyl groups tend not to inhibit crystal growth and could accelerate the calcium phosphate precipitation reaction [82].

Suitable acids for this type include citric acid, glycolic acid, malic acid, tartaric acid, succinic acid, and maleic acid. Once the initial setting has been completed, the cement’s calcium phosphate components continue to interact, resulting in more stable end products [83].

**Table 1:** Previous studies on soil improvement using calcium phosphate compounds.

Precipitation Source	Ca and P Source	Soil Type	Addition		Chemical Concentration	Treatment Duration (Days)	Precipitation Type	Crystal Morphology	UCS (kPa)	Reference
Microbially mediated reaction between calcium phosphate compounds										
Acidotolerant urease-producing bacteria (*Staphylococcus saprophyticus*)	Feed bone meal	Cracked stone	-		1:1 (Ca/Urea)	2	Hydroxyapatite	Rod-like and plate-like microparticles	ND	[68]
Dimorphic phytase-active (*Arxula adeninivorans*)	Calcium phytate	Glass beads	-		-	3	Monetite, whitlockite and hydroxyapatite	Needle-like crystals	ND	[69]
Soil-derived bacteria	Ca^2+^ and PO_4_^3−^	Alluvial topsoil	-		1:1 (Ca^2+^/PO_4_^3−^)	5	Hydroxyapatite and calcite	Bacteria-like hydroxyapatite and rhombohedral calcite	ND	[70]
Enzymatically mediated reaction between calcium phosphate compounds										
Acid urease (*Nagapshin*)	Bone meal powder (Cows)	Toyoura sand	-		0.25:1 (Ca/Urea)	16	Brushite	Amorphous-like	1620	[71]
Phytase enzyme	Sodium glycerophosphate (SGP)	Lead-zinc tailings pond sample	Mg^2+^		1.5 M SGP	3	Newberyite and lead phosphate	ND	2700	[73]
Enzymatically mediated reaction between calcium phosphate compounds										
Urease (watermelon seeds (*Citrullus vulgaris*) extract)	Chemicals (DPP and CA)	Toyoura sand	-		1.5:0.75 M (DAP:CA) + urease (solid—liquid ratio of 0.005)	28	ND	Specific crystal structure could not be identified	125.6	[72]
Chemical reaction between calcium phosphate compounds										
Chemicals (diammonium phosphate (DAP) and calcium acetate (CA))		Toyoura sand	10% of tricalcium phosphate (TCP) powder		1.5:0.75 (DAP:CA)	28	ND	Whisker-like crystal	261.4	[74,75]
Chemicals (dipotassium phosphate (DPP) and CA)		Toyoura sand	10% of scallop shell (SS) powder		1.2 M: 0.6 M (DPP:CA)	56	ND	Not clearly identify a crystal formation among sand particles	156.9	[76,77]
Chemicals (DAP and CA)		Toyoura sand	Phosphate powders	10% of tricalcium phosphate (TCP) powder	1.5:0.75 M (DAP:CA)	28	ND	Whisker-like crystal	250	[78]
				1% magnesium phosphate (MgP) powder		14		Numerous 10-μm-long crystals	75	
			Carbonate powders	5% calcium carbonate (CC) powder		56		Unified structures of sand particles and CPC precipitation	250	
				1% magnesium carbonate (MgC) powder		28		Numerous 10-μm-long crystals without unification with sand particles	110	
Chemical reaction between calcium phosphate compounds										
Chemicals (DAP and CA)		Toyoura sand	-		1.5:0.75 M (DAP:CA)	14	Hydroxyapatite	Whisker-like crystal	63.5	[79]
Chemicals (DAP and calcium nitrate (CN))					1.0 M:0.5 M (DAP:CN)	14		Plate-like crystals	20	
Chemicals (DAP and CA)		Toyoura sand	-		1.5:0.75 M (DAP:CA)	28	Hydroxyapatite	Whisker-like crystal	87.6	[80]
Reaction between calcium and carboxylic acids										
Chemicals (DAP and CA)		Toyoura sand	Extract from agricultural alkaline and acidic soil (source of microorganisms) and amino acid source (asparagine (Asn), glutamine (Gln) and glycine (Gly))		1.5:0.75 M (DA:CA) + 0.1 M amino acid	28	ND	Whisker-like crystal	50–100	[83]
Chemicals (DAP and CN)					1.0 M:0.5 M (DAP: CN) + 0.1 M amino acid			Plate-like crystals		

Note: ND—Not determined; Toyoura sand—it is a clean standard silica sand without inclusions, with particles ranging in size from 0.1 mm to 0.5 mm, which is available commercially in Japan and used for different experiments [84].

**Figure 5:**
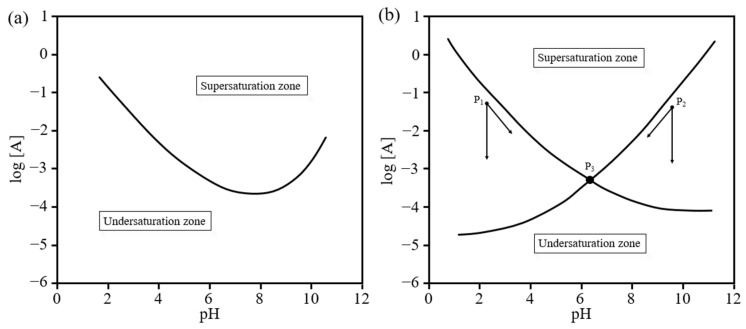
Solubility diagram of a general mineral compound (**a**) and the relative position of isotherms of two hypothetical compounds in the system (**b**). log [A] is the ion concentration of hypothetical compound A, pH—pH of the solution [85].

For both types of CPCs, the precipitation of minerals from an aqueous solution occurs when the aqueous solution becomes oversaturated with the mineral compound (powder phase of calcium phosphates). When the supersaturation and undersaturation levels of the mineral compound and the aqueous phase thermodynamically equate to each other, precipitation occurs (Figure 5a). When equilibrium is not reached, the dissolution–precipitation process will continue until the pH and composition reach a singular point. Table 2 shows the range of pH stability in aqueous solutions for different CPCs. At the equilibrium point, they will react with each other and the product will precipitate (Figure 5b) [85]. 

Some of the CPCs cannot be precipitated from an aqueous solution. These compounds can be obtained as a product of a high temperature, solid-phase reaction. Among these compounds are α-tricalcium phosphate, β-tricalcium phosphate, and tetracalcium phosphate (Table 2, b). The only CPCs that can harden at ambient temperature as a result of the reaction between two calcium phosphates and an aqueous solution are hydroxyapatite, calcium-deficient hydroxyapatite, octacalcium phosphate, and dicalcium phosphate dihydrate [86].

**Table 2:** A list of the calcium orthophosphates and their major properties [87,88].

Ca/P Ratio	Compound	Abbreviation	Formula	Solubility at 25 °C, g/L	pH Stability Range in Aquatic Solutions at 25 °C
**0.5**	Monocalcium phosphate monohydrate	MCPM	$\mathrm{Ca}{\left(H_{2}{\mathrm{PO}}_{4}\right)}_{2}\cdot H_{2}O$	~18	0.0–2.0
**0.5**	Monocalcium phosphate anhydrate	MCPA	$\mathrm{Ca}{\left(H_{2}{\mathrm{PO}}_{4}\right)}_{2}$	~17	a
**1.0**	Dicalcium phosphate dihydrate	DCPD	${\mathrm{CaHPO}}_{4}\cdot 2H_{2}O$	~0.088	2.0–6.0
**1.0**	Dicalcium phosphate anhydrate	DCPA	${\mathrm{CaHPO}}_{4}$	~0.048	a
**1.33**	Octacalcium phosphate	OCP	${\mathrm{Ca}}_{8}{\left({\mathrm{HPO}}_{4}\right)}_{2}{\left({\mathrm{PO}}_{4}\right)}_{4}\cdot 5H_{2}O$	~0.0081	5.5–7.0
**1.5**	α-tricalcium phosphate	α-TCP	$\alpha -{\mathrm{Ca}}_{3}{\left({\mathrm{PO}}_{4}\right)}_{2}$	~0.0025	b
**1.5**	β-tricalcium phosphate	β-TCP	$\beta -{\mathrm{Ca}}_{3}{\left({\mathrm{PO}}_{4}\right)}_{2}$	~0.0005	b
**1.2–2.2**	Amorphous calcium phosphate	ACP	${\mathrm{Ca}}_{x}{\left({\mathrm{PO}}_{4}\right)}_{y}\cdot {\mathrm{nH}}_{2}O$	c	5–12
**1.5–1.67**	Calcium-deficient hydroxyapatite	CDHA	${\mathrm{Ca}}_{10-x}{\left({\mathrm{HPO}}_{4}\right)}_{x}{\left({\mathrm{PO}}_{4}\right)}_{6-x}{\left(\mathrm{OH}\right)}_{2-x}\;$ (0<x<1)	~0.0094	6.5–9.5
**1.67**	Hydroxyapatite	HA	${\mathrm{Ca}}_{10}{\left({\mathrm{PO}}_{4}\right)}_{6}{\left(\mathrm{OH}\right)}_{2}$	~0.0003	9.5–12
**2.0**	Tetracalcium phosphate	TTCP	${\mathrm{Ca}}_{4}{\left({\mathrm{PO}}_{4}\right)}_{2}O$	~0.0007	b

a—stable at temperature over 100 °C. b—these CPCs are not able to be precipitated from aquatic solutions. c—cannot be measured accurately.

CPC complex structures are composed of a CaP-based powder and a liquid phase that react chemically when mixed to form a crystalline solid [67]. The setting mechanism of calcium-phosphate compounds begins with the dissolution of salts in the solution, resulting in the release of HPO_4_^2−^ and Ca^2+^ ions and, eventually, the deposition of CPCs [89]. Precipitation is controlled by pH and the concentration of calcium and phosphate in solution [90]. The pH of the solution can be controlled by catalyzing urea hydrolysis with plant-derived or bacterial urease [91]. The deposition of crystalline hydroxyapatite, the final product of the reaction between calcium and phosphate salts, depends on the Ca/P atomic ratio in solutions and/or microenvironments, where the presence of large amounts of dissolved Ca^2+^ is preferable for better precipitation [70,92].

**Figure 6:**
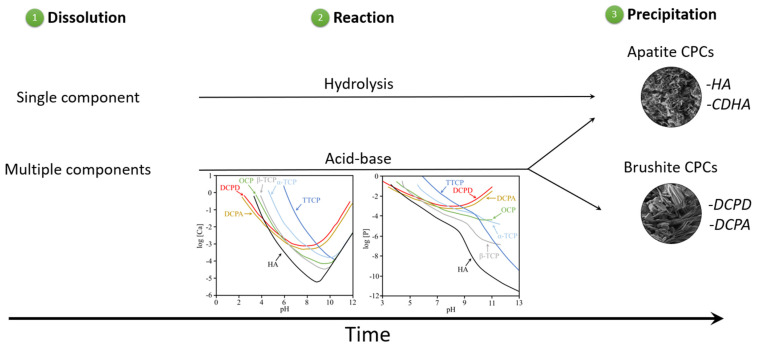
A representation of the calcium phosphate precipitation.

CPCs may be classified into two types: apatite CPCs (final product is HA or CDHA) and brushite CPCs (DCPD or DCPA) (Figure 6). The solubility of the CaP precursor compounds and the pH at which the reaction was performed, both affect the type of the final product [67].

**Figure 7:**
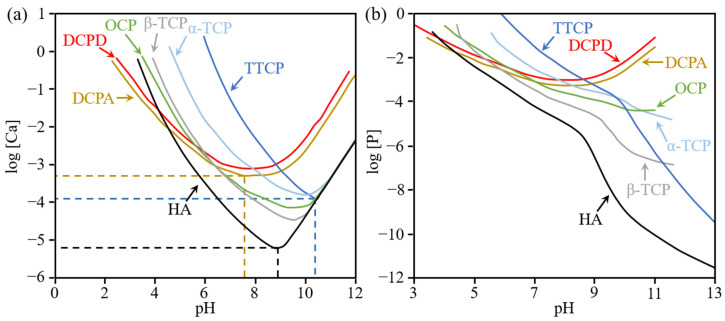
The solubility of the calcium phosphate compounds as a function of the Ca (**a**) and P (**b**) concentration dependence on pH [87,93].

**Figure 8:**
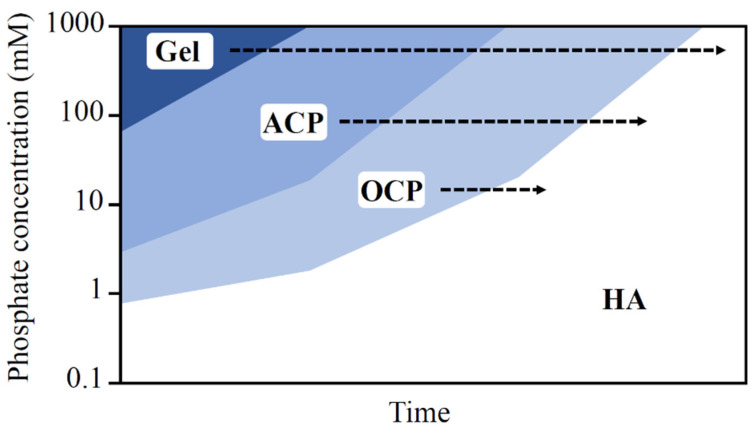
The formation, stability, and hydrolysis of calcium phosphates as a function of phosphate concentration (log (P)) in solutions at neutral pH [87].

Calcium phosphate compounds can be divided into 11 different types, depending on the Ca/P ratio (Table 2). The formation of each of them highly depend on the pH of the solution and time (Figure 7 and Figure 8).

CPCs are readily soluble in an acidic environment and tend to precipitate at a pH range from 7.5 to 10.5. For precipitation HA, the most soluble and stable compound, the pH of the solution should be around 9 (Figure 7). Overall, alkali conditions are required for the complete precipitation of CPCs. HA forms directly in solutions with low P concentrations, when the concentration of P is greater than 100 mM, a colloidal gel-like and viscous calcium phosphate is formed. Over time, this phase softens to ACP, hydrolyzed to OCP and eventually converts to hydroxyapatite (Figure 8). The reaction rate is influenced by pH and the calcium and phosphate ion concentrations. CPCs solidify due to the self-setting mechanism (Table 3) [94], which are highly dependent on the phosphate ion concentration and pH.

#### 3.1.1. CPCs from Calcium Phytate

**Figure 9:**
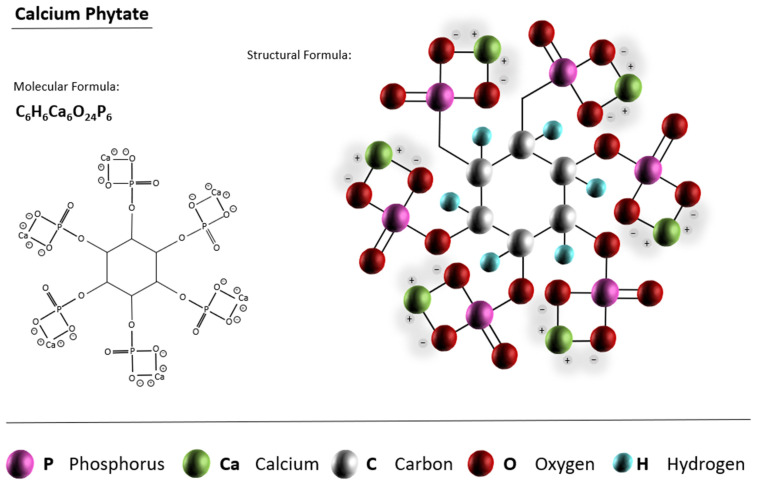
The representation of the calcium phytate structure.

Phytates are phytic acid salts or myo-inositol hexakidydihydrogen phosphate [95]. The phytate is capable of forming compounds with many cations such as Fe^2+^, Mg^2+^, Cu^2+^, Ca^2+^, Zn^2+^, limited by the biodistribution of the minerals [96]. This compound can be found in barley, dry beans, corn, cottonseeds, oats, maize, peanut, peas, rape seed, rice, sunflower, wheat, soy, rye, sesame, etc. [97,98,99]. It is estimated that annually, up to 51 million metric tons of phytate accumulate in the soil, making it a readily available resource [100]. Figure 9 schematically illustrates the structure of phytate.

**Figure 10:**
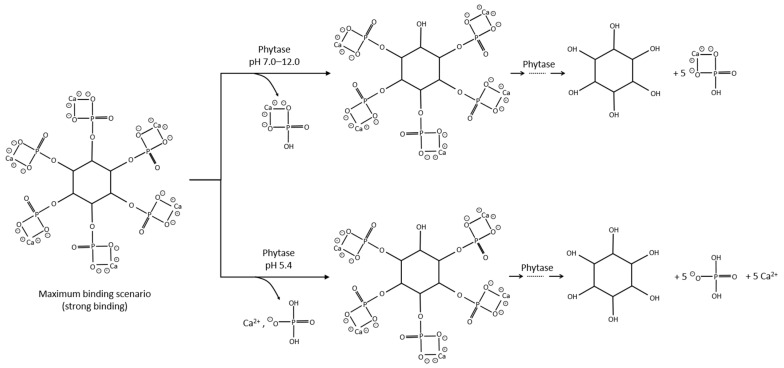
Schematic representation of the hypothetical reaction between calcium phytate and phytase [101].

Phytase is one of the first recorded enzymes, which liberates inorganic phosphate from organophosphorus compounds [97]. Phytase is able to trigger phytate dephosphorylation in stages, releasing calcium ions into solution where it can react with orthophosphates to form CPCs such as monetite (CaHPO_4_), whitlockite [Ca_9_(Mg, Fe^2+^)(PO_4_)_6_HPO_4_], and hydroxyapatite [Ca_5_(PO_4_)_3_OH], (Figure 10) [27,101,102].

Numerous microorganisms contain an enzyme called phytase, which can liberate phosphate groups from phytate [103]. Phytases are secreted by different groups of microbes, yeast, and bacteria [69]. Among the yeasts, *Aspergillus niger*, *Aspergillus ficuum*, *Aspergillus fumigatus*, and *Saccharomyces cerevisiae* are widely adopted strains for commercial phytase production [98,99]. Various bacteria such as *Escherichia coli, Bacillus subtilis, Klebsiella terringa*, and *Lactobacillus* sp. are known to produce phytase [95,98,99]. *A. adeninivorans* are among the few yeasts with high phytase activity and cell mass release capable of metabolizing phytate as the single source of carbon and phosphate. Its optimal pH and temperature is between 4.5–5.0 and 60 °C, respectively [103]. *A. adeninivoransis* is an especially perspective source of microbial phytase and therefore for calcium phosphate precipitation.

CPC precipitation through the phytase decomposition of phytate was first implemented for the biocementation of glass beads and showed the formation of monetite, whitlockite, and hydroxyapatite after 3 days [69]. The application of phytate and the phytase enzyme also has the potential to fix radionuclides and metal contaminants in groundwater systems by co-precipitating these pollutants with calcium phosphate [69,73]. Previous results have shown that the application of phytate, phytase, and magnesium ions to the lead-zinc tails resulted in the deposition of MgHPO_4_(H_2_O)_3_ (newberite) and Pb_9_(PO_4_)_6_ (lead phosphate), strengthening the tails by bonding the tail particles and filling the pores. The strength of the treated sample of tails could reach 2.7 MPa at a reactant concentration of 1.5 M after 3 days of treatment [73].

Since this method uses phytase, which is completely safe (used in the food industry [104]) and has no by-products such as ammonium, it is environmentally friendly to enhance the soil. However, there is one disadvantage: large amounts of calcium phytate must be applied to the soil due to its low solubility, increasing the cost of this method [27]. Since organophosphorus compounds are expensive, this method is more suitable for soils rich in insoluble phosphorus [21].

#### 3.1.2. Microbially Induced CPCs Precipitation

In order to precipitate the CPCs, the pH of the medium needs to increase from 4.5 to 8. One way to achieve this involves the usage of acidotolerant urease-producing bacteria, another is to apply pure acidotolerant urease.

Below are the formulas for the hydroxyapatite hydrolysis precipitation reaction using acidotolerant bacteria (Equations (7)–(10)) and acid urease (Equation (11)).

i. Monetatite (dicalcium phosphate anhydrate) precipitation by acidotolerant bacteria [27]

Precipitation of monetatite due to the reaction between monocalcium phosphate anhydrate, urea, and acidotolerant bacteria:

(7) $$Ca{\left(H_{2}PO_{4}\right)}_{2}+CO{\left(NH_{2}\right)}_{2}+H_{2}O\;\overset{acidotolerant\;bacteria}{\to }CaHPO_{4}↓+CO_{2}\;+{\left({\mathrm{NH}}_{4}\right)}_{2}{\mathrm{HPO}}_{4}\;$$

ii. Hydroxyapatite precipitation by acidotolerant bacteria [9,27,68]

Precipitation of hydroxyapatite due to the reaction between monocalcium phosphate anhydrate, urea, and acidotolerant bacteria:

(8) $$5Ca{\left(H_{2}PO_{4}\right)}_{2}+8CO{\left(NH_{2}\right)}_{2}+8H_{2}O\;\overset{acidotolerant\;bacteria}{\to }Ca_{5}{\left(PO_{4}\right)}_{3}\left(OH\right)↓\;+\;2{\mathrm{NH}}_{4}{\mathrm{HCO}}_{3}+6{\mathrm{CO}}_{2}+7{\left({\mathrm{NH}}_{4}\right)}_{2}{\mathrm{HPO}}_{4}$$

Precipitation of hydroxyapatite due to the increase in the pH from the enzymatic hydrolysis of urea and the reaction between the calcium and phosphate ion source:

(9) $$0.5\mathrm{CO}{\left({\mathrm{NH}}_{2}\right)}_{2}+1.5H_{2}O\;\overset{\mathrm{acidotolerant}\;\mathrm{bacteria}}{\to }{\mathrm{NH}}_{4}{}^{+}+{\mathrm{OH}}^{-}+0.5{\mathrm{CO}}_{2}$$

(10) $$10{\mathrm{Ca}}^{2+}+6H_{2}{\mathrm{PO}}_{4}{}^{-}+14{\mathrm{OH}}^{-}\;\overset{\;}{\to }{\mathrm{Ca}}_{10}{\left({\mathrm{PO}}_{4}\right)}_{6}{\left(\mathrm{OH}\right)}_{2}↓+12H_{2}O\;$$

iii. Hydroxyapatite precipitation by acidic urease [68]

The precipitation of hydroxyapatite in situ is due to an increase in the pH by acid urease and the reaction between dicalcium phosphate anhydrate and the calcium ion source:

(11) $$4{\mathrm{Ca}}^{2+}+6{\mathrm{CaHPO}}_{4}+8{\mathrm{OH}}^{-}\;\overset{\mathrm{acid}\;\mathrm{urease}}{\to }{\mathrm{Ca}}_{10}{\left({\mathrm{PO}}_{4}\right)}_{6}{\left(\mathrm{OH}\right)}_{2}↓+6H_{2}O\;$$

For hydroxyapatite formation to be stable, the initial pH of the solution must be below 5. This makes the presence of acid resistant urease-producing bacteria or raw acid resistant urease one of the most important factors affecting the course of the reaction. The bacterium or urease is most active in an acidic environment, capable of raising the pH from 4.5–5.0 to 7.5–8.0, resulting in the subsequent precipitation of hydroxyapatite [52].

While this method is more environmentally friendly than MICP and EICP, as low pH is able to precipitate CPCs, ammonium formation still occurs during the reaction. In order to use this method in field tests, the ammonium problem must be solved. Alternative ways to overcome the ammonium issue with this method are to use dead bacterial cells/low-cost enzyme or chemical methods to bind the ammonium inside the soil.

### 3.2. Prospects and Merits

CPCs have great potential for field applications in the future. Although its use for soil enhancement started in the last decade, it has demonstrated its environmental friendliness and viability. CPCs are non-toxic [80]; originally, they began to be used in medicine and dentistry, which itself proves their complete harmlessness to the environment.

Along with pure chemicals, which have been used for decades to precipitate hydroxyapatite, animal bones and seafood shells can be used as a source of calcium and phosphate for CPCs [71]. The calcium and phosphate salts required for the precipitation of CPCs can be obtained from industrial wastes such as animal bones. They are an inexpensive alternative to chemicals, and are readily available and easily commercially available.

Compared to calcium, which is abundant, phosphorus (P) is a limited and reducing resource [105]. Due to the prospective shortage of phosphate resources in the future, this deficiency must be addressed [106,107]. CPCs can address the challenge of this resource scarcity. After soil improvement, a slab consisting of soil, rock, and CPCs can be re-excavated and turned into an agricultural fertilizer, thereby avoid wasting a valuable resource [90,108].

Atmospheric pollution by ammonia, the main limitation of MICP and EICP applications in the field, can be controlled by using calcium phosphate precipitation [24]. Since the precipitation reaction takes place in the pH range below 8, this automatically reduces the emission of ammonium into the atmosphere by a factor of 10. Replacing the bacteria and urease with a reactant capable of controlling pH will reduce the cost and pollution. One such method that can be considered is the electro-mediated precipitation of calcium phosphate. In this method, electricity is used to raise the pH of the medium, which leads to the formation of calcium phosphates in the presence of calcium and phosphorus ions in the system. First, at a low pH, an amorphous phase is formed, which then transforms into the most stable crystal-line phase, hydroxyapatite [109].

The use of calcium phosphate can be an alternative method for soil reinforcement and has advantages over MICP and EICP. In other words, it has a great potential as a material for research and development of an innovative material that can replace the use of cement as a soil enhancing material and ultimately reduce the emission of CO_2_ into the atmosphere.

Along with the predominant advantages, CPCs has significant drawbacks. The greatest disadvantage is the cost of this method. Since calcium phosphates have a low solubility, it is necessary to inject larger amounts of solution containing calcium and phosphorus salts in order to achieve a measurable strength of the soil. The second disadvantage is the low mechanical properties of this material [89]. As of today, the highest strength value obtained using this method is 2.7 MPa [73], which is more than half of the UCS of EICP improved soil.

## 4. Conclusions

In this review article, the new method for soil improvement—CPC—was discussed. A comparison with existing techniques and their disadvantages was made. The process of obtaining CPC was shown and the studies that are available at the moment were examined.

The method of soil improvement using CPCs is a relatively recent technique. It was first used for soil improvement in 1999, and since 2010, it has been investigated in greater depth. This methodology remains new and unstudied, but there are already various studies proving the prospects of this technology. The following methods have been used for soil improvement with CPCs: microbially mediated CPC precipitation (using acidotolerant urease producing bacteria, soil derived bacteria and acidotolerant yeast); enzymatically mediated CPC precipitation (using acid urease, phytase enzyme and urease from plants); chemical reactions between CPCs (diammonium phosphate, calcium acetate, dipotassium phosphate and calcium nitrate).

From previous studies, the highest UCS value was 2.7 MPa using phytase enzyme for CPC precipitation. Compared to MICP and EICP, where the maximum UCS was 12.4 MPa and 6.5 MPa, respectively, this result is competitive since urease was used.

CPC precipitation requires a minimum pH increase in the range of 4.5–8. However, the pH of the solution must be less than 6.5 to prevent free ammonia. Using MICP and EICP methods, the pH rises as high as 9, which leads to the conversion and release of up to 40% ammonium in gaseous form. When CPCs are used, the gaseous emission of ammonium can be reduced from 40% to 6%. Nevertheless, these compounds can precipitate, even at a pH of less than 6.5, which completely solves the ammonia problem. Thus, this method has great potential for application in the field.

The next point that needs to be pointed out is that CPCs are non-toxic and were originally used in medicine. Based on the above-mentioned statement, once the soil is improved, the CPCs can be re-excavated and used as a fertilizer for agriculture.

The main disadvantage of this technology is the high cost of treatment. Since a large amount of calcium and phosphorus needs to be pumped into the soil, this makes the price of this method higher. However, when using another method to raise the pH instead of bacteria or urease, the price can be reduced by 98%, thereby equalizing the cost increase due to the materials needs.

## 5. Future Perspectives

Analyzing all of the above-mentioned, in order to improve the existing techniques of soil treatment and ensure an environmentally friendly engineering approach, some problems have to be solved in the future:

i. Alternative resources for calcium and phosphate need to be found. Most of the chemicals that are available on the market are aimed at the medical field. These are costly and are not suitable for soil improvement on a large scale. Animal bones are an excellent option, however, since the natural hydroxyapatite needs to be separated from the fatty inclusions, which requires acids, it is not an ideal candidate for use outside the laboratory.
ii. For the deposition of CPCs, the atomic concentration of phosphorus and calcium in the system and the pH of the medium must be taken into account. In field applications, it is challenging to control the pH of the soil in such a small range to obtain the desired compound. Introducing bacteria may simplify this, although the problem of ammonium contamination in the environment will emerge. To overcome such an issue, it is necessary to investigate new methods of incorporating CPCs into the soil and control the parameters accurately.
iii. In a long-term perspective, the low durability problem should be solved. The investigation of the effect of the application of CPCs to the soil could have an impact on the strength of the soil. Since this is a novel approach to ground reinforcement, the combination of the MICP and EICP research results over many decades and the combination of them for applying with CPCs can reveal its potential and make this material an ideal analogue for cement in the future.
iv. In order to improve the existing soil stabilization methods using CPCs, the problem of ammonium must be addressed. Using bacteria or urease in this method, 6% ammonium in a gaseous form contaminates the atmosphere. Therefore, in future studies, this should be taken into account and correlated by adding different additives or by changing the parameters of the precipitation reactions.

Once these problems are solved, using CPCs will allow the soil to be improved without the use of cement or cement-containing additives, which in turn will reduce carbon dioxide emissions into the atmosphere. Improving this method by making it completely ecologically friendly can lead to a zero footprint in some areas of engineering. CPCs have a series of advantages over MICP and EICP, which makes them preferable for future research and applications.

## Figures and Tables

**Table 3:** The hydrolysis of calcium phosphate compounds [78].

Compound	Hydrolysis
**Monocalcium phosphate**	$5\mathrm{Ca}{\left(H_{2}{\mathrm{PO}}_{4}\right)}_{2}\;\cdot H_{2}O\;\overset{\;\;\;\;\;\;\;\;\;\;\;\;\;}{\to }{\;\mathrm{Ca}}_{5}{\left({\mathrm{PO}}_{4}\right)}_{3}\mathrm{OH}+7H_{3}{\mathrm{PO}}_{4}+4H_{2}O\;$
**Dicalcium phosphate**	$5{\mathrm{CaHPO}}_{4}+H_{2}O\;\overset{\;\;\;\;\;\;\;\;\;\;\;\;\;}{\to }{\;\mathrm{Ca}}_{5}{\left({\mathrm{PO}}_{4}\right)}_{3}\mathrm{OH}\;+2H_{3}{\mathrm{PO}}_{4}$
**Octacalcium phosphate**	$5{\mathrm{Ca}}_{8}{\left({\mathrm{PO}}_{4}\right)}_{6}H_{2}\;\cdot 5H_{2}O\;\overset{\;\;\;\;\;\;\;\;\;\;\;\;\;}{\to }8{\mathrm{Ca}}_{5}{\left({\mathrm{PO}}_{4}\right)}_{3}\mathrm{OH}+6H_{3}{\mathrm{PO}}_{4}+17H_{2}O$
**Tricalcium phosphate**	$5{\mathrm{Ca}}_{3}{\left({\mathrm{PO}}_{4}\right)}_{2}+3H_{2}O\;\overset{\;\;\;\;\;\;\;\;\;\;\;\;\;}{\to }\;3{\mathrm{Ca}}_{5}{\left({\mathrm{PO}}_{4}\right)}_{3}\mathrm{OH}\;+{\;H}_{3}{\mathrm{PO}}_{4}$
**Hydroxyapatite**	${\mathrm{Ca}}_{5}{\left({\mathrm{PO}}_{4}\right)}_{3}\mathrm{OH}$
**Tetracalcium phosphate**	$3{\mathrm{Ca}}_{4}{\left({\mathrm{PO}}_{4}\right)}_{2}O+3H_{2}O\;\overset{\;\;\;\;\;\;\;\;\;\;\;\;\;}{\to }\;2{\mathrm{Ca}}_{5}{\left({\mathrm{PO}}_{4}\right)}_{3}\mathrm{OH}\;+2\mathrm{Ca}{\left(\mathrm{OH}\right)}_{2}$

## Data Availability

No new data were created or analyzed in this study. Data sharing is not applicable to this article.
